# Fluorinated Nanosized Zeolitic-Imidazolate Frameworks as Potential Devices for Mechanical Energy Storage

**DOI:** 10.1021/acsami.4c09969

**Published:** 2024-08-23

**Authors:** Eder Amayuelas, Judit Farrando-Perez, Alexander Missyul, Yaroslav Grosu, Joaquin Silvestre-Albero, Carolina Carrillo-Carrión

**Affiliations:** † Centre for Cooperative Research on Alternative Energies (CIC EnergiGUNE), Basque Research and Technology Alliance (BRTA), 01510 Vitoria-Gazteiz, Spain; ‡ Laboratorio de Materiales Avanzados, Departamento de Química Inorgánica-Instituto Universitario de Materiales, Universidad de Alicante, 03690 San Vicente del Raspeig, Spain; § Institute of Chemistry, University of Silesia, 40-006 Katowice, Poland; ∥ Institute for Chemical Research (IIQ), CSIC-University of Seville, 41092 Sevilla, Spain; # CELLS—ALBA Synchrotron, 08290 Cerdanyola del Vallès, Barcelona, Spain

**Keywords:** zeolitic-imidazolate frameworks, fluorination, water intrusion/extrusion, mechanical energy storage/dissipation

## Abstract

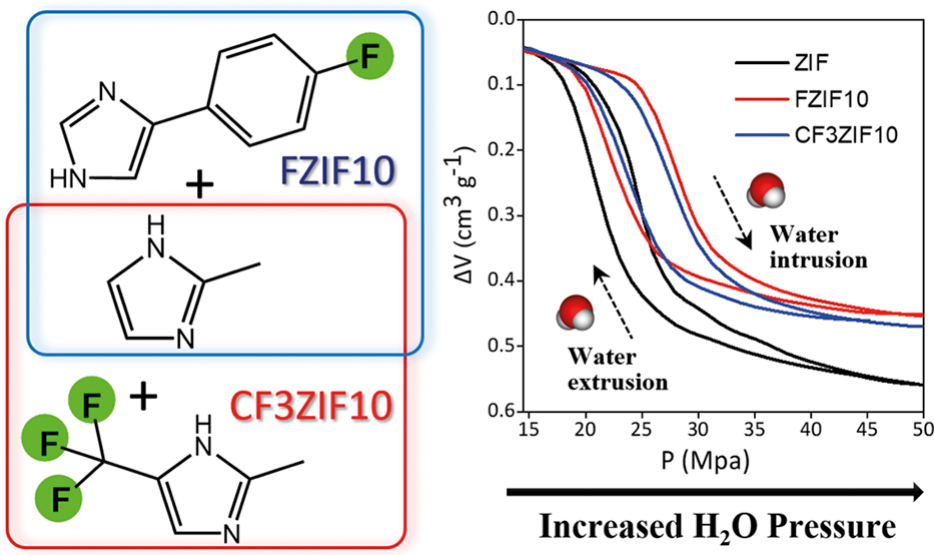

Fluorination is one of the most efficient and universal strategies to increase the hydrophobicity of materials and consequently their water stability. Zeolitic-imidazolate frameworks (ZIFs), which have limited stability in aqueous media and even lower stability when synthesized on a nanometric scale, can greatly benefit from the incorporation of fluorine atoms, not only to improve their stability but also to provide additional properties. Herein, we report the preparation of two different fluorinated ZIFs through a simple and scalable approach by using mixed ligands [2-methylimidazole, as a common ligand, and 4-(4-fluorophenyl)-1*H*-imidazole (*monofluorinated* linker) or 2-methyl-5-(trifluoromethyl)-1*H*-imidazole (*trifluorinated* linker) as a dopant], demonstrating the high versatility of the synthetic method developed to incorporate different fluorine-containing imidazole-based ligands. Second, we demonstrate *for the first time* that these nanoscale fluorinated ZIFs outperform the pristine ZIF-8 for water intrusion/extrusion, i.e., for storing mechanical energy via forced intrusion of nonwetting water due to the improved hydrophobicity and modified framework dynamics. Moreover, we also show that by varying the nature of the F-imidazole ligand, the performance of the resulting ZIFs, including the pressure thresholds and stored/dissipated energy, can be finely tuned, thus opening the path for the design of a library of fluorine-modified ZIFs with unique behavior.

## 1 Introduction

Metal–organic frameworks (MOFs) are porous materials consisting of metal ions or clusters linked through organic ligands.^1^ MOFs have recently attracted great interest in the fields of energy storage, conversion, and dissipation, mainly due to two key features: (i) their porous nature, with regular porosity and high accessible area and (ii) their high chemical and structural tunability, which allows access to MOFs with very diverse physicochemical properties depending on the specific application.^2,3^ Despite these benefits, many types of MOFs also suffer important limitations, such as the lack of stability in humid conditions and in aqueous media, as well as the limited framework resistance under high pressures.^4−6^ This is, for example, the case of zeolitic-imidazolate frameworks (ZIFs). ZIFs consist of tetrahedrally coordinating divalent metal cations coordinated to imidazole-based linkers, thus giving rise to zeolite-like frameworks with several topologies.^7^ ZIFs are characterized by a large surface area (much higher than zeolites and porous silicas), high thermal stability, and an acceptable tolerance to elevated pressures. However, chemical stability, especially in the presence of water and phosphates, is their Achilles’ heel.^8^ These stability issues are especially critical when working with ZIFs at the nanoscale (i.e., nanosized ZIFs or ZIF-based nanoparticles), since the decomposition kinetics under unfavorable conditions are much faster in smaller particles.^9^ However, many other properties of ZIFs are generally maximized in nano- versus microsized particles.^10^ Consequently, the search for stabilization strategies for the ZIF framework while maintaining the nanosize is highly desirable.

For energy-storage purposes, MOFs are in principle ideal candidates as heterogeneous lyophobic systems (HLS) because they combine unique characteristics.^11−16^ These include high energy density, fast response, reversibility, and low environmental impact. Moreover, ZIFs present also interesting intrusion/extrusion characteristics due to their unique framework flexibility and negative compressibility.^17,18^ The operation of HLSs relies on understanding the interactions between water as a nonwetting liquid and a hydrophobic porous material. The process of H_2_O intrusion/extrusion under hydrostatic pressure is crucial for controlled energy storage and/or conversion. When pressure is applied (mechanical energy), water intrudes into the pores against its natural tendency, storing potential energy (solid/liquid interfacial energy) within the porous structure. Upon pressure release, water is extruded spontaneously, and the interfacial energy is converted back into mechanical energy.^19−21^ The extent of energy absorbed and released defines the characteristics of the device, i.e., either a molecular spring (small or preferably negligible hysteresis loop) or an energy dissipation system (large hysteresis loop). Recent studies described in the literature have already addressed the good performance of several ZIFs in water intrusion/extrusion experiments, e.g., ZIF-8,^22^ ZIF-67,^23^ Co/Zn-ZIF,^12^ ZIF-71,^23^ demonstrating comparable performance to mesoporous grafted silica and zeolites, despite their subnanometer sized pores.^11,22,24^ All studies reported to date on ZIFs point out the promising performance of these materials, not only in mechanical energy storage and dissipation but also in related applications such as nanotriboelectric generators,^21^ column chromatography,^25,26^ separation,^27^ purification,^28^ etc. Despite the significant advances in this field during the past decade, the limited long-term stability of ZIFs in water due to the hydrolysis of the Zn–N bonds, and subsequent dissolution of the ZIF framework, significantly hinders the actual potential of ZIFs under real operation conditions (e.g., immersion in water for long periods of time and repetitive intrusion/extrusion cycles under elevated pressures), thus precluding their effective translation to industrial scenarios. It is worth noting here that new approaches to increase water stability in ZIFs for such particular applications must consider aspects such as scalability, sustainability, and cost-effectiveness.

In recent years, there has been an increased interest in the development of new MOF structures through the incorporation of a highly electronegative fluorine group, thus providing highly stable materials with novel physicochemical properties associated with the strongly polarized bonds. The fluorination strategy can be directed to the incorporation of fluorinated inorganic units based on metallic (e.g., transition metals) or semimetallic (e.g., fluorinated inorganic anions) clusters or to the incorporation of fluorinated organic linkers.^29,30^ Interestingly, these fluorine-decorated MOFs exhibit peculiar properties in a wide range of applications, e.g., specific interactions of the fluorine groups with CO_2_, improved hydrophobicity, and improved selectivity for unsaturated hydrocarbons due to the enhanced affinity, among others.^31−35^ However, to the best of our knowledge, fluorinated MOFs have never been applied for mechanical energy storage and/or dissipation. It is worth noticing that high-pressure water intrusion/extrusion is a highly demanding stability test for any MOF,^36^ which is relevant for other applications mentioned above.

Constructing MOFs from multiple components (i.e., “mixed-linker”, “hybrid”, or “multivariate” MOFs) is one straightforward and effective strategy to tune the MOFs’ properties, resulting in the improvement of one specific property, the combination of additional functionalities in one single material, and even in the appearance of synergistic effects with application in cooperative catalysis and gas adsorption. In the literature, there are diverse examples of ZIFs synthesized with combinations of different imidazolate linkers. This can be achieved either via one-pot synthesis routes or through postsynthetic modifications, this latter by replacing linker molecules or incorporating other linkers into MOFs using solvent assisted linker exchange or solvent assisted linker incorporation routes, respectively.^37,38^ Generally, one-pot synthesis leads to greater efficiency in the incorporation of the secondary linker as well as to a more homogeneous distribution of it throughout the entire structure, but this route is not always possible, as it will depend notably on the similarity between the linkers. In some cases, dramatic changes including crystal-to-crystal transformations are observed through the multivariate route or upon postsynthetic solvent-assisted modifications, resulting in MOFs with very different properties. In other cases, however, it is possible to synthesize MOFs with linker compositions intermediate between two single-linker MOFs, showing a continuous and gradual tuning of any functional property such as adsorption. Therefore, the most convenient strategy for preparing mixed-linker MOFs must be planned specifically according to the desired effect. Although one-pot synthesis of mixed-linker ZIFs containing two or more linkers with different connectivity and symmetry has been reported,^39,40^ it is still synthetically challenging to achieve a good control in the homogeneity of ZIF particles, which is specifically relevant in some energy applications, as mentioned above.

With the above in mind, herein, we propose the synthesis of fluorinated ZIF-8 nanoparticles through a one-pot mixed-linkers approach to easily introduce fluorine atoms along the whole ZIF framework and explore their impact on their chemical stability and, even more interestingly, on their water intrusion/extrusion performance. In this way, we intent to investigate *for the first time* the influence of fluorination on the water intrusion/extrusion volume and energy storage/dissipation capacity of nanosized ZIF-8 compared to the nonfluorinated counterpart, which may be the key to the rational design of more efficient MOF-based energy applications and applications where MOFs are subjected to high-pressure liquid media such as separation.

## 2 Experimental Section

### 2.1 Materials

All reagents were obtained from commercial sources and were used without further purification. These reagents were zinc nitrate hexahydrate [Zn(NO_3_)_2_·6H_2_O; Merck, 98%], 2-methylimidazole (MeImz; Sigma-Aldrich), 4-(4-fluorophenyl)-1*H*-imidazole (FImz; BLD Pharmatech; 98%), 2-methyl-5-(trifluoromethyl)-1*H*-imidazole (CF3Imz; BLD Pharmatech; 95%), methanol (MeOH; Fischer Scientific, 99.8%), and deuterated methanol (CD_3_OD; Merck, ≥99.8 atom % D).

### 2.2 Synthesis of Fluorinated ZIFs

The synthesis of fluorinated ZIFs was carried out following an optimized protocol developed by some of us, consisting in the mixture of the imidazole linkers (MeImz and FImz or CF3Imz) and the Zn precursor in a solvent mixture MeOH/H_2_O (1:1), stirring for 5 min, and leaving the mixture undisturbed for allowing the crystals’ growth during 24 h. The experimental conditions were optimized in order to achieve homogeneous nanoparticles (in shape and size), achieve quantitative incorporation of the fluorine-imidazole linkers, and maximize the reaction yield. Fluorinated ZIF-8 nanoparticles will be labeled as FZIF10 (prepared using MeImz and FImz) and as CF3ZIF10 (prepared using MeImz and CF3Imz), with 10 corresponding to the mol % of the fluorinated linker added in the synthesis. The pristine, nonfluorinated, ZIF-8 will be labeled as ZIF. The detailed procedures were as follows:

#### 2.2.1 FZIF10 or CF3ZIF10 Synthesis

MeImz (0.864 mmol), fluorinated Imz-ligand (0.096 mmol, either FImz or CF3Imz, 10 mol % of total ligand content), and Zn(NO_3_)_2_·6H_2_O (0.06 mmol) were dissolved in 3 mL of MeOH/H_2_O (1:1) under magnetic stirring (350 rpm) at room temperature (RT) for 5 min. After that, the mixture was left undisturbed for 24 h. A pale yellowish-colored turbidity or whitish turbidity in the case of FZIF10 or CF3ZIF10, respectively, was appearing over time in a gradual manner, indicative of the formation of the particles. After 24 h, the resulting particles were collected by centrifugation and washed 3 times with methanol in order to remove the excess of precursors and remaining water solvent trapped in the pores. The final purified particles were dried overnight at 80 °C and further activated thermally at 120 °C for 6 h.

#### 2.2.2 ZIF Synthesis

MeImz (0.960 mmol) and Zn(NO_3_)_2_·6H_2_O (0.06 mmol) were dissolved in 3 mL of MeOH/H_2_O (1:1) under magnetic stirring (350 rpm) at RT for 5 min and left then undisturbed for 24 h. The purification and activation process were the same as described above.

#### 2.2.3 Scale-Up Synthesis

Upscaling trials were performed by increasing the amount of precursors and the solvent proportionally. An 10× up-scale did not affect the quality of the obtained fluorinated particles, as discussed below for FZIF10 particles. Similar results were obtained for the control ZIF and CF3ZIF10 particles. Once the possibility of scaling up the procedure was checked, we proceeded with the samples prepared in large-scale for the complete structural analysis and the following water intrusion/extrusion studies.

### 2.3 Characterization Techniques

#### 2.3.1 Scanning Electron Microscopy

Scanning electron microscopy (SEM) images were acquired with a HITACHI S4800 field emission microscope operating at 2 kV in secondary electron and backscattered electron modes. Samples were prepared by drying a diluted suspension of the particles in methanol on a silicon wafer substrate.

#### 2.3.2 Powder X-ray Diffraction

X-ray analysis of the crystalline powder samples was performed using a Bruker D8-Advanced diffractometer. X-ray radiation of Cu Kα (0.15406 nm) was used, and the measurements were recorded in 2θ steps of 0.02° in the 5–50° range.

#### 2.3.3 Synchrotron X-ray Powder Diffraction and Rietveld Refinement

Synchrotron X-ray powder diffraction (SXRPD) data were collected at the MSPD beamline of the ALBA synchrotron light source (Spain) using a MYTHEN2 detector (λ = 0.4138 Å, refined using the NIST 640d standard).^48^ Experiments were performed at 20 °C in the capillary reaction cell (fused silica capillary; inner diameter, 0.7 mm; outer diameter, 0.85 mm), using the activated ZIFs. A GSAS-II software package was used for Rietveld refinement of the obtained data.^49^ Due to the relatively low amount of dopant ligands and their structural similarity to 2-methylimidazole, explicit addition of the dopants to structure refinements had a negligible effect on the fitting quality. Consequently, diffraction data of both FZIF10 and CF3ZIF10 were refined using the pure ZIF-8 structure model. Briefly, the original 2-methylimidazole linkers in the structure were randomly substituted with fluorinated linkers (see CIF files in the Supporting Information 2, Supporting Information 3, Supporting Information 4). Positions of atoms belonging to imidazole rings coincide for 2-methylimidazole and fluorinated linkers. Interatomic distances and angles of the substituents in the fluorinated linkers were close to their values in similar molecules. Corresponding bond and angle restraints were applied during the refinement, and occupancy of the atoms belonging to the substituents in the fluorinated linkers was fixed at 0.05 (10 mol % substitution). Since linkers are not symmetrical, 2 equiv orientations were possible.

#### 2.3.4 Nuclear Magnetic Resonance Spectroscopy

^1^H nuclear magnetic resonance (NMR) and ^19^F NMR spectra were recorded on a 400 MHz Bruker Avance III HD spectrometer. ^1^H NMR analyses were performed to determine the actual amount of the dopant linker (fluorinated ligands, FImz or CF3Imz) incorporated into the frameworks. For that, the fluorinated ZIF particles were acid-digested with D_2_SO_4_/CD_3_OD, and the ^1^H NMR spectrum of the resulting mixture was recorded.

#### 2.3.5 Dynamic Light Scattering

Measurements were performed using a Malvern Zetasizer Nano ZSP instrument equipped with a 10 mW He–Ne laser operating at a wavelength of 633 nm. A diluted suspension of the particles was loaded into a quartz cuvette, and measurements were taken after an equilibration step of 2 min. Size distribution results were generated by averaging 3 consecutive measurements (12 data runs each).

#### 2.3.6 N_2_ Physisorption Analysis

N_2_ isotherms (−195 °C) were obtained using a home-built manometric equipment designed and constructed by the “Advanced Materials Laboratory—LMA” group. Before the adsorption measurements, the samples were outgassed under vacuum at 150 °C for 24 h. The apparent surface area was calculated from the BET equation in the pressure range *p*/*p*_0_ ∼ 0.01–0.2 (being *p*_0_ the saturation pressure). Micropore volume was calculated using the Dubinin–Radushkevich (DR) equation, while the total pore volume was estimated at *p*/*p*_0_ ∼ 0.90.

#### 2.3.7 H_2_O Physisorption Analysis

H_2_O adsorption isotherms (25 °C) were obtained using a home-built manometric equipment designed and constructed by the LMA group and now commercialized by Anton Paar as VSTAR. Before the adsorption measurements, the samples were outgassed under vacuum at 150 °C for 24 h.

### 2.4 Water Intrusion/Extrusion Experiments

Water intrusion/extrusion tests were carried out by means of water porosimetry. Typically, each ZIF material was mixed with pure water and encapsulated in a flexible and hermetic polymeric capsule prior to testing. An AutoPore IV 9500 porosimeter (Micromeritics Instrument Corporation, Norcross, USA) was used for the compression tests, where the penetrometer was evacuated to a pressure less than 7 Pa, followed by filling with mercury to 50 MPa. For the stability tests, all the materials were subjected to five H_2_O consecutive water intrusion/extrusion cycles for 24 h, following the conditions previously described. Once the cycles were finished, the materials were taken out from the capsule and dried at 60 °C overnight prior to powder X-ray diffraction (PXRD) to characterize the crystallinity after the H_2_O intrusion/extrusion process.

## 3 Results and Discussion

### 3.1 Design and Synthesis of Fluorinated ZIFs

With the selection of appropriate experimental conditions (see the Experimental Section), we were able to successfully prepare two types of fluorinated ZIF-8 nanoparticles in a high yield (>80%) via a one-pot mixed linker synthetic method under mild and sustainable conditions, i.e., at RT and using a mixture of water/methanol (1:1) as solvent; note that methanol is classified as a green solvent.^41^ The primary linker was 2-methylimidazole (MeImz), whereas the dopant linkers were 4-(4-fluorophenyl)-1*H*-imidazole (FImz) and 2-methyl-5-(trifluoromethyl)-1*H*-imidazole (CF3Imz). This selection of fluorine-containing imidazole-based ligands allowed us to investigate the effect of the number of fluorine atoms and ligand structure in the synthesis of the fluorinated ZIF particles and their subsequent performance. In order to have a significant fraction of F-linkers in the framework but without compromising the topology and crystallinity in the final F-doped ZIF particles, the amount of doped linker in the precursor solution was set at 10 mol % (being MeImz/FImz or MeImz/CF3Imz, 9:1 molar ratio), giving rise to nanoparticles denoted as FZIF10 and CF3ZIF10, respectively. This could be considered to be an upper limit for the successful functionalization of ZIF-8. In fact, in an attempt to increase the fluorine content to 20 mol % in the FZIF sample, we observed that crystallinity was largely lost, obtaining very small and amorphous particles. It is hypothesized that the bulky fluorophenyl group in position 4 of the imidazole linker could probably greatly distort the tetrahedral units of Zn(Imz)_2_ due to steric effects among FImz groups, thus compromising the crystallinity at high dopant levels. Based on our previous experience on ZIFs, the ligand-to-metal ratio was fixed in all cases to 16 by using an excess amount of imidazole-linkers. The idea was to favor the deprotonation of the imidazole molecule, which is the key to guarantee fast nucleation with slow crystal growth, thus leading to the formation of nanosized particles. Control ZIF-8 nanoparticles (denoted as ZIF) were prepared using the same experimental conditions but without the addition of dopant F-linkers.

Using this one-pot synthetic strategy (also known as de novo synthesis), the mechanism of formation implies the incorporation of dopant F-based organic linkers during the crystallization process. It was found that the use of different F-linkers affected the kinetics of the growth of the crystals, these processes being notably faster in the case of FZIF10 particles as revealed by the quick appearance of turbidity during the synthesis (compared to CF3ZIF10 and control ZIF particles). The synthesis time was extended to 24 h to ensure maximum yields, which were 88 and 81% for FZIF10 and CF3ZIF10, respectively (on the basis of the amount of the zinc source used), and also to achieve a quantitative incorporation of the dopant F-linkers, as determined by ^1^H NMR analysis (see Supporting Information Figures S1–S5). The actual dopant amounts in the frameworks were determined through ^1^H NMR after acid digestion of the particles, obtaining values of 10 and 9.6 mol % for FZIF10 and CF3ZIF10, respectively. These results clearly revealed the successful incorporation of the F-linkers into the framework in a quantitative manner under the optimized synthetic conditions. Here, it is important to note that the selection of the solvent is critical. Indeed, a mixture of water/methanol (1:1) led to quantitative doping, whereas when using pure methanol for the synthesis, the amount of FImz incorporated in FZIF10 particles was only 4 mol % (compared to the 10 mol % added). This finding is in line with previously reported data showing a low incorporation of dopant linkers in similar mixed-linker ZIFs prepared in methanol.^42^

### 3.2 Influence of Fluorination in the Structural Properties

**Figure 1:**
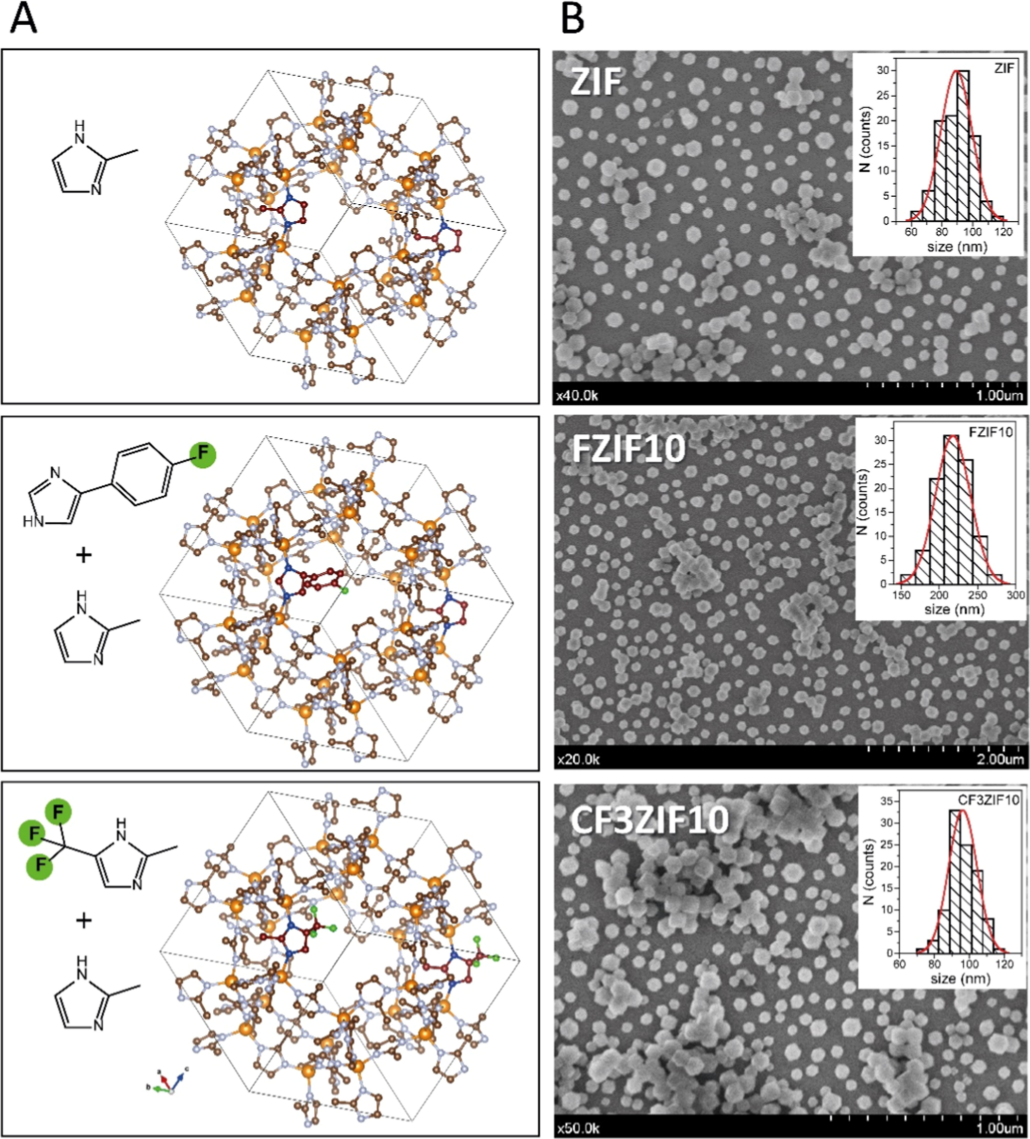
(A) Illustration of the 3D structures of the different ZIFs evaluated. (B) Representative SEM micrographs of the as-prepared ZIF, FZIF10, and CF3ZIF10 particles. Inset: Histograms of the particle size distribution (idealized as spherical particles) as determined from SEM micrographs.

**Figure 2:**
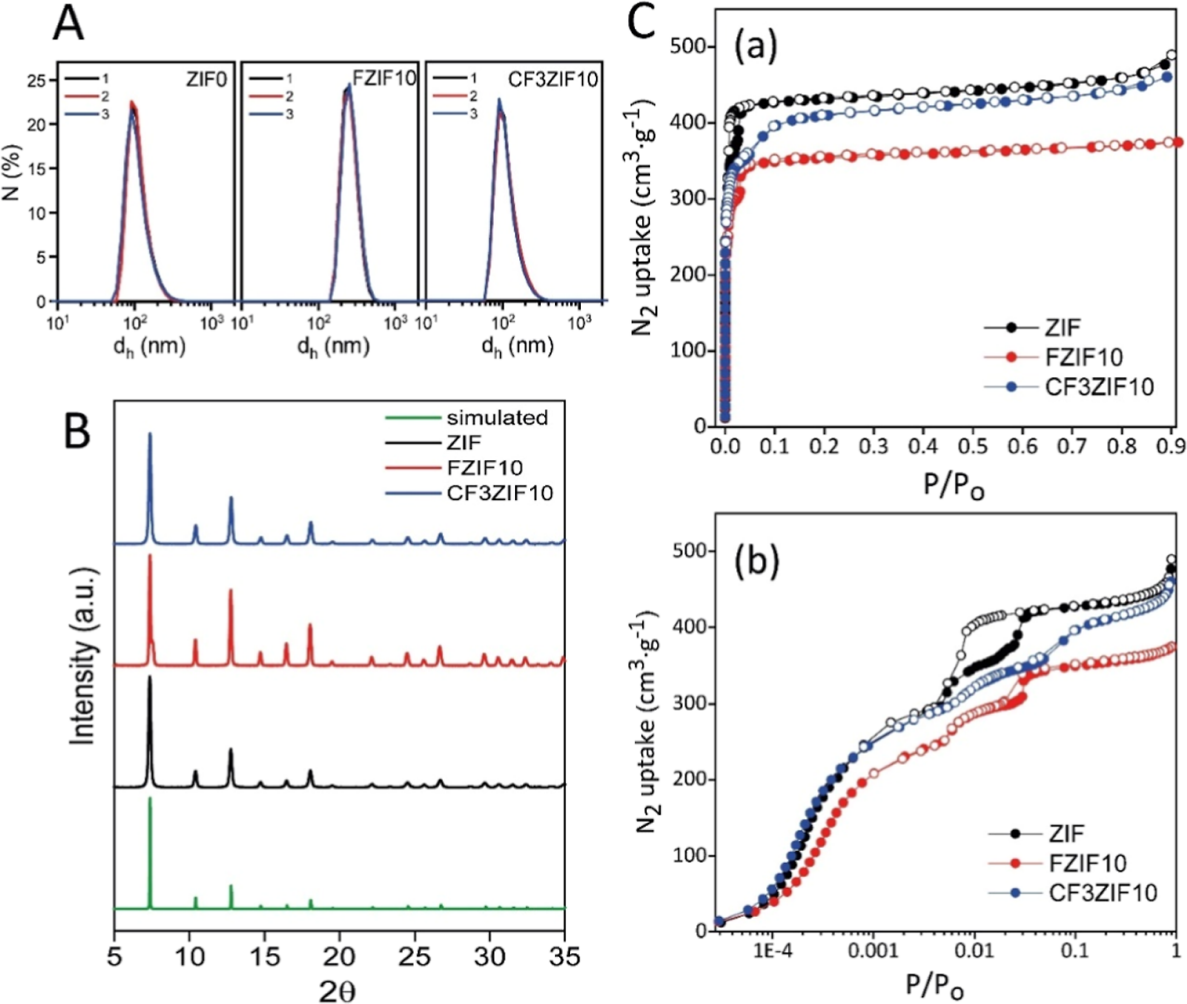
(A) DLS size distribution by number of the as-prepared ZIF, FZIF10, and CF3ZIF10 particles dispersed in methanol (*n* = 3 measurements are shown). (B) PXRD patterns of the as-prepared particles, including the simulated pattern for ZIF-8. (C) N_2_ adsorption/desorption isotherms for the different ZIFs evaluated at −195 °C in (a) linear and (b) logarithmic scales.

The particle morphology (size and shape) of the as-prepared particles was examined by using SEM, as shown in Figure 1. The theoretical structures of the three ZIFs evaluated are included for the sake of clarity (Figure 1A). Both fluorinated FZIF10 and CF3ZIF10 particles presented the prototypical rhombic dodecahedron shape, equal to that observed for control ZIF. According to the SEM images, the average particle sizes were determined to be 217 ± 23, 96 ± 8, and 89 ± 10 nm, for FZIF10, CF3ZIF10, and ZIF, respectively (Figure 1B). These values agree with their hydrodynamic sizes in solution, as determined by dynamic light scattering (DLS) (Figure 2A, Table S1). The notable larger size of the FZIF10 particles was expected due to their faster growth. It is important to know that in all cases, the particles were quite homogeneous (i.e., narrow size distributions and low PdI in DLS), indicative of the good synthetic control in the crystal formation. Again, due to the faster growth in FZIF10, its PdI value was slightly larger. Keeping in mind the importance of the particle quality for some energy-related applications,^43^ such as the one investigated here, but also that scalability is one of the biggest challenges for the translation of MOF-based application to the industry, we tried to scale-up the optimized synthetic method. Interestingly, we did not find significant changes in the structure and yield when doing a 10× synthesis (Figure S6), demonstrating that our method meets in principle some key requirements (i.e., simple, scalable, and sustainable) for further practical uses.

Regarding the crystalline structure of the fluorinated particles, both were found to be highly crystalline as determined by PXRD (Figure 2B), showing the same diffraction pattern as the control ZIF particles. The observed broadening of some peaks in the CF3ZIF10 particles may be attributed to the smaller particle size compared to the FZIF10 sample. This result suggests that both fluorinated ZIFs adopted the same framework structure of the parent ZIF-8, in agreement the synchrotron X-ray diffraction measurements. Rietveld refinement (Figure S7) confirms the similarity in unit cell parameters for the three ZIFs evaluated, i.e., ZIF, *a* = 17.0082(5) Å, FZIF10, *a* = 17.0136(4) Å, and CF3ZIF10, *a* = 17.0087(7) Å.^44^ SPXRD patterns also confirm the smaller crystal size of CF3ZIF10 compared to the other samples (average crystalline sizes obtained from XRD data are 150 nm for ZIF; 190 nm for FZIF10; and 110 nm for CF3ZIF10). These values are similar to those estimated from SEM data, thus confirming that most particles are single crystals. The textural properties of the fluorinated ZIFs were evaluated using N_2_ adsorption measurements at cryogenic temperatures (Figure 2C). The isotherm of the nonfunctionalized ZIF resembles that of the commercial sample (Basolite Z1200; Figure S8) with the characteristic steps at *p*/*p*_0_ ∼ 5 × 10^–3^ and *p*/*p*_0_ ∼ 1.3 × 10^–2^, attributed to the swinging of the imidazolate linker upon adsorption, with the associated accommodation of additional nitrogen molecules.^44−46^ Interestingly, the smaller crystal size in the synthesized ZIF particles (vs commercial Basolite) is reflected in a shift of the swinging of the imidazolate linker to higher *p*/*p*_0_ values, a wider hysteresis loop, and the presence of some capillary condensation in the interstitial spaces at high relative pressures.^45^ The BET surface areas of these samples were 1678 m^2^ g^–1^ for ZIF and 1570 m^2^ g^–1^ for Basolite Z1200 (Table S2). Despite the similarity in the crystal structure of the fluorine-functionalized ZIFs, the presence of functional groups in the crystal structure gives rise to important differences in the nitrogen isotherms, including the gate-opening effect. In the specific case of the –CF_3_ group (CF3ZIF10 particles), the N_2_ isotherm exhibits a similar adsorption performance to pristine ZIF at low relative pressures (inner cavities should not be affected by the –CF_3_ groups at the pore aperture) and preserves the initial characteristic step at *p*/*p*_0_ ∼ 5 × 10^–3^. However, the second step in the N_2_ isotherm is shifted to higher pressures, ca. *p*/*p*_0_ ∼ 5 × 10^–2^, followed by a completely reversible hysteresis loop. This behavior anticipates the presence of two different linker domains in the ZIF structure, the swinging of the bulkier –CF_3_, and surrounding MImz linkers being restricted to higher pressures. For the particles containing the monofluorinated ligand (FZIF10), the N_2_ adsorption isotherm also reflects the two characteristic steps at *p*/*p*_0_ ∼ 5 × 10^–3^ and *p*/*p*_0_ ∼ 2 × 10^–2^, but associated with a narrower hysteresis loop and a smaller amount of N_2_ adsorbed both at low relative pressures and at saturation. Apparently, the bulkier FImz linker limits the adsorption performance of the FZIF10 particles due to the decreased volume of the inner cavity (in agreement with the micropore volume, Table S2) and limits the extent of the second step (restricted swinging). BET surface area for the functionalized particles ranges from 1545 m^2^ g^–1^ for CF3ZIF10 down to 1327 m^2^ g^–1^ for FZIF10.

Water adsorption isotherms at 25 °C (Figure S9) confirm the hydrophobic nature of all ZIFs evaluated with a limited adsorption capacity (<0.8 mmol/g). A closer look at these isotherms shows that the hydrophobicity follows the order ZIF < CF3ZIF10 < FZIF10. Despite the presence of three fluorine atoms in the CF3Imz linker, the water adsorption capacity of FZIF10 is slightly lower than that of CF3ZIF10, most probably due to the combined presence of a fluorine atom and an aromatic group (both hydrophobic) and the hindered accessibility for H_2_O by the bulkier FImz linker.

### 3.3 Impact of Fluorination in Chemical Stability

**Figure 3:**
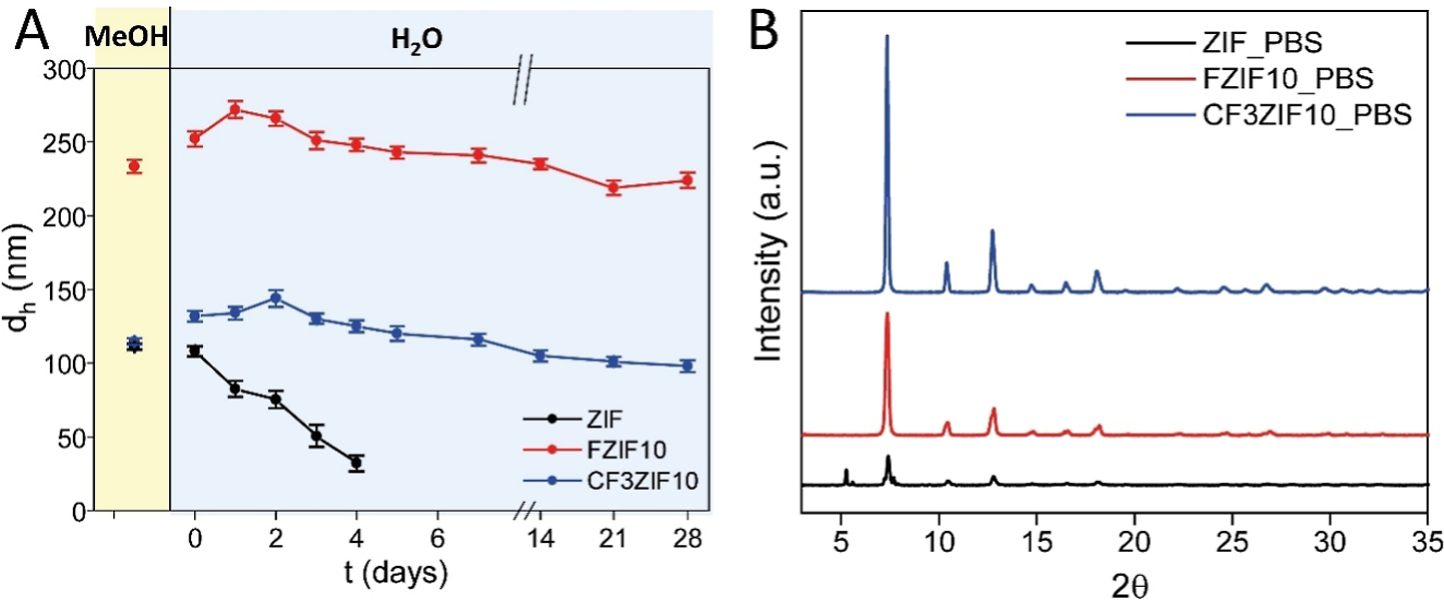
(A) Hydrodynamic diameter (*d*_h_) of the as-synthesized particles dispersed in either methanol and Milli-Q water at *t* = 0, and colloidal stability over time (up to 4 weeks) of the particles dispersed in water, as determined by DLS. (B) PXRD patterns of the as-synthesized particles after 48 h of incubation in PBS (0.1 M, pH = 7.4).

To investigate further the impact of the fluorination on the stability of the particles in water, the changes in the hydrodynamic diameter of the samples dispersed in Milli-Q water were monitored over time. As shown in Figure 3A, in contrast to ZIF, both fluorinated particles show an exceptional stability in water even being at very low concentrations (20 μg/mL), confirming that the presence of fluorine atoms in the structure improves the stability of the ZIF in water due to the increased hydrophobic character of the framework. Expanding the study to the evaluation of chemical stability, more unfavorable conditions were tested, specifically the stability in the presence of phosphates. It is well-known that phosphate ions can attack the uncoordinated Zn ions on the particle surface, promoting the generation of surface defects and the further dissolution of the particles.^8^ To study the effect of phosphates, FZIF10, CF3ZIF10, and ZIF particles were incubated with a PBS solution (0.1 M, pH = 7.4) for 48 h. After this time, the particles were collected by centrifugation, washed twice with methanol, and dried. PXRD of these PBS-treated samples (Figure 3B) indicated that fluorination seems to protect the framework from the interaction/attack of phosphate ions or at least making the structure less sensitive to phosphate-induced degradation. It is also worth noting that CF3ZIF10, with a higher density/concentration of fluorine atoms, preserved better the crystallinity after PBS treatment, which may be attributed to the higher local electronegativity around those fluorine atoms and the consequent better repulsion of the phosphate anions.

### 3.4 Evaluation of Fluorinated ZIFs for Mechanical Energy Storage and Dissipation

**Figure 4:**
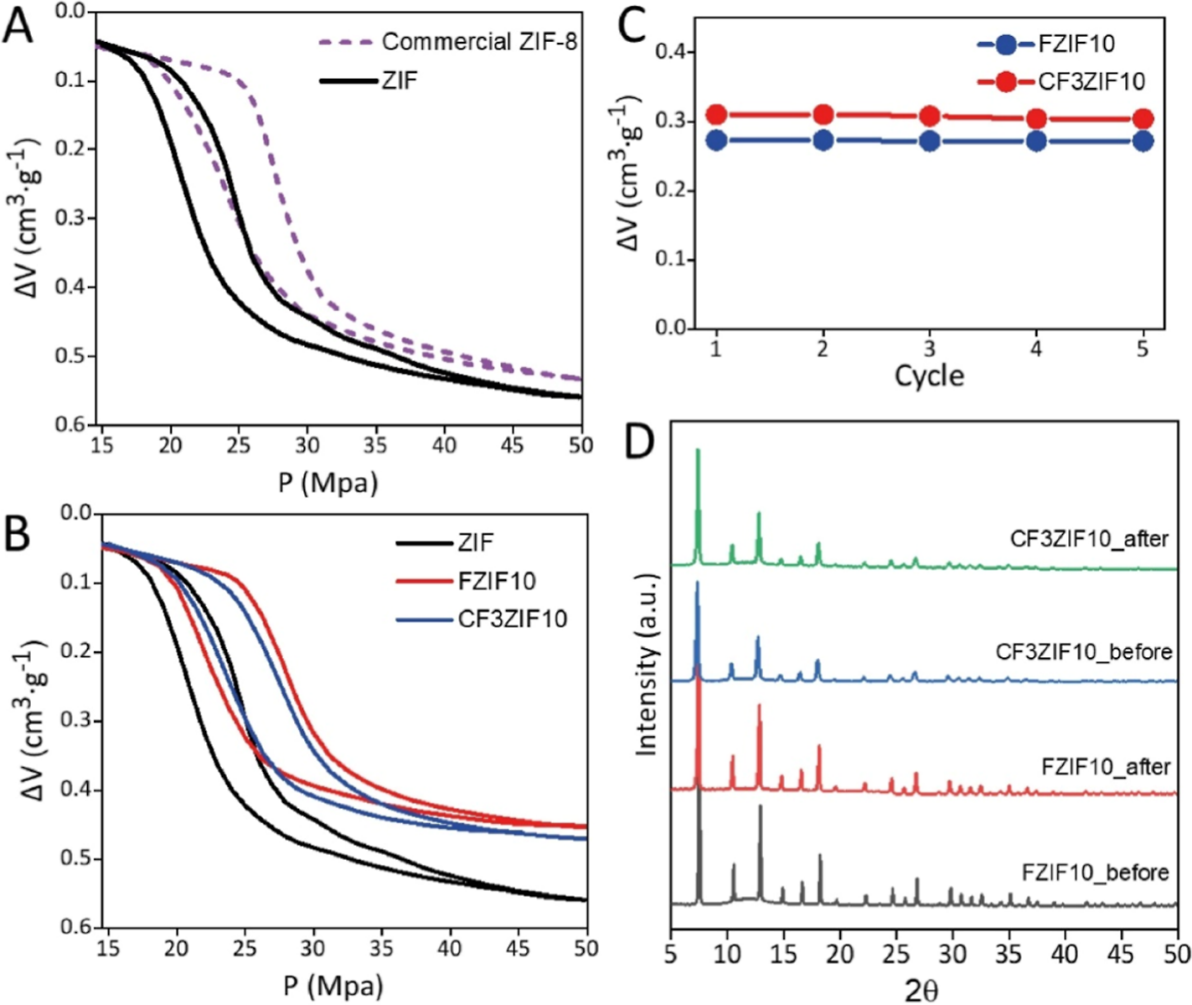
(A) *PV*-isotherms of commercial ZIF-8 (purple dashed line) and ZIF (black line). (B) *PV*-isotherms of ZIF (black line), FZIF10 (blue line), and CF3ZIF10 (red line). See derivatives of the intrusion and extrusion branches of *PV* isotherms for all samples in Figure S12. (C) Evolution of *V*_int_ during 5 cycles of FZIF10 (blue line) and CF3ZIF10 (red line). (D) Comparison of PXRD patterns of the fluorinated particles (FZIF10 and CF3ZIF10) before and after five H_2_O intrusion/extrusion cycles.

With these fluorinated ZIFs in hand, the H_2_O intrusion/extrusion performance was evaluated. In a first step, the energy storage and dissipation performance of the as-synthesized ZIF particles was measured and compared to that of commercial ZIF-8 (Basolite Z1200) as a reference. As shown in Figure 4A, both pressure–volume (*PV*) isotherms differ in intrusion and extrusion pressures (*P*_int_ and *P*_ext_), and also in intrusion volume (*V*_int_). As-synthesized ZIF particles exhibit a *P*_int_ of 24.5 MPa, i.e., 2.8 MPa lower than the commercial ZIF-8, in good agreement with previous results reported in the literature.^15^ A critical difference between these two materials is the crystal size (compare Figures 1B and S10). ZIF shows a unimodal crystal size distribution with a mean value of ∼89 nm, while commercial ZIF-8 presents a broader size distribution with much larger crystals. As previously reported, the crystal size in ZIF-8 plays a key role defining *P*_int_ for values below 200 nm, i.e., lowering ZIF-8 crystal size decreases *P*_int_.^47^ Following this statement, crystals below 100 nm also reduce *V*_int_. However, as shown in Figure 4A, nanosized ZIF presents a higher *V*_int_ than commercial ZIF-8. This counterintuitive behavior could be related to the quality of the synthesized material, in agreement with textural parameters (larger micropore volume for ZIF versus commercial ZIF-8; Table S2). Taking into account the optimal synthesis protocol resulting in highly crystalline material, with a unimodal nanosized distribution and euhedral shape of ZIF (as shown in Figure 1B), this would imply a higher quality material than commercial ZIF-8, i.e., including fewer structural defects and/or less amorphous phase contributions. The higher crystal quality in ZIF particles may also be responsible for the higher symmetry of the hysteresis loop between intrusion and extrusion cycles, e.g., contrary to the commercial ZIF-8, in the nanosized ZIF particles crystal domains respond to the external stress in a narrower pressure window. This performance is also reflected in a smaller energy dissipation between intrusion and extrusion for the high quality ZIF particles compared to the commercial ZIF-8 (1.55 vs 1.65 J/g), i.e., ZIF is a higher quality molecular spring.

Similar H_2_O porosimetry tests were performed for the other two fluorinated ZIFs: FZIF10 and CF3ZIF10 (Figure 4B). Both materials demonstrate clear differences in the intrusion/extrusion performance compared to the nonfunctionalized ZIF particles (Table 1). Functionalized ZIFs show relatively high intrusion pressures compared to pristine ZIF, due to their hydrophobic nature: *P*_int_ values of 27.4 MPa, for FZIF10, and 28.4 MPa, for CF3ZIF10. Also, the intruded volume is affected after the fluorination of the ZIFs, the lowest value (0.27 cm^3^ g^–1^) corresponding to sample FZIF10, followed by sample CF3ZIF10 (0.31 cm^3^ g^–1^), and finally the ZIF sample (0.37 cm^3^ g^–1^). This tendency perfectly agrees with the micropore volume calculated from the nitrogen adsorption isotherms (Table S2), *V*_int_ being defined by the nature (size and shape) and quantity of the dopant incorporated. Additionally, the pressure difference between intrusion and extrusion was calculated to identify the performance of these ZIFs for mechanical energy storage, e.g., systems with a small hysteresis in the *PV* isotherm. These values range from 23% in the nonfluorinated ZIF to 35 and 24% for FZIF10 and CF3ZIF10, respectively. These values are in the range of many zeolites (e.g., LTA zeolite—15.2%; chabazite zeolite—16.2%) and similar to or smaller than those reported in the literature for other ZIFs (e.g., ZIF-8—24.8%; ZIF-67—38.3%; ZIF-71—57.7%).^11^ Whereas some ZIFs can be considered mechanical energy dissipators (more than 50% of energy dissipated in ZIF-71), our ZIF nanoparticles are able to store mechanical energy in a “nearly reversible” way (Table 1), i.e., these can be considered molecular springs. In fact, the dissipated energy is relatively small for all samples evaluated (below 25%), except FZIF10, most probably due to the hindered rotation of the bulkier FImz linker. A closer look at Table 1 nicely describes that, upon fluorination, the dissipated energy (J/g) increases (ca. 18%) for FImz as a linker (FZIF particles, 1.83 J/g) and decreases (12%) for CF3Imz as a linker (CF3ZIF10 particles, 1.36 J/g). Furthermore, both fluorinated ZIFs keep the symmetry in the intrusion/extrusion cycle, in agreement with the high quality of the synthesized particles. Overall, our results demonstrate that the fluorination of ZIFs can be a promising approach to finely modulate the mechanical energy behavior of ZIFs in HLSs in one direction or another (toward molecular-springs or shock-absorber) by an appropriate and rationally designed functionalization of the MOF structure. The nature and amount of the functionalities (size, shape, and physicochemical properties) can be tuned to provide a proper dissipator or a molecular spring, depending on the final application, thus opening the gate to apply these materials in energy devices devoted to compensate the temporal mismatch between energy production and demand (for instance, in renewable energy sources), or as a mechanical dissipators for shocks.

**Table 1:** Intrusion/Extrusion Parameters for ZIF, FZIF10, and CF3ZIF10^a^

sample	*V*_int_/*V*_ext_ (cm^3^ g^–1^)	*P*_int_/*P*_ext_ (MPa)	*W*_int_ (J/g)	*W*_ext_ (J/g)	dissipated energy (J/g)^b^	Δ*W* hysteresis (%)^c^
ZIF-8 (Basolite)	0.33/0.33	27.3/24.2	6.118	4.463	1.65	27
ZIF	0.37/0.36	24.5/20.1	6.638	5.084	1.55	23
FZIF10	0.27/0.27	27.4/22.4	5.339	3.507	1.83	35
CF3ZIF10	0.31/0.31	28.4/23.7	5.528	4.168	1.36	24.5

a Commercial ZIF-8 is included for the sake of comparison.

b Estimated from the integrated area for intrusion and extrusion.

c Δ*W* = (*W*_int_ – *W*_ext_)/*W*_int_ × 100.

The observed results in terms of *P*_int_ and *V*_int_ underlie different hypotheses:

(i) *The reduction of pore size and pore aperture.* The inclusion in ZIF-8 structure of 10 mol % of fluorinated ligand with different morphology and molecular size does not affect the crystal structure, as both fluorinated materials keep the SOD topology (Figure 2B). However, both secondary/dopant ligands (FImz and CF3Imz) are bulkier than the primary one (MeImz), thus reducing the pore size and pore aperture. Under these conditions, higher pressures are needed for water to intrude the hydrophobic pores, and therefore a greater *P*_int_.
(ii) *Different hydrophobicity of each ZIF.* The inclusion of fluorinated ligands in ZIF-8 structure increases the hydrophobicity of hybrid ZIFs compared to pristine MeImz-based ZIF, which play a key role in the higher *P*_int_. This hypothesis is reinforced by the observed trend for all ZIFs (Figure 4B, Table 1), where *P*_int_ increases as follows ZIF < FZIF10 ≈ CF3ZIF10. The similar value for FZIF10 and CF3ZIF10 complies with their hydrophobic character due to the fluorine functionalities and the aromatic group, in the case of FImz ligand. However, the direct comparison between both samples is not straightforward due to their different crystal size (FZIF10 > CF3ZIF10) and the different nature of the ligand incorporated (presence or not of the bulkier aromatic group).
(iii) *Reduction of pore volume.* As confirmed by N_2_ adsorption isotherms, the micropore volume decreases with the size of the ligand (ZIF > CF3ZIF10 > FZIF10). A lower micropore volume available to accommodate guest H_2_O molecules implies lower *V*_int_, which fits with the observed trend.

In light of these results, the possibilities offered by hybrid systems in which it is possible to accommodate ligands with different characteristics represent a new way to grant new properties or improve well-known MOFs, allowing them to maintain those characteristics essential for their dissipation performance and mechanical energy storage.

### 3.5 Stability of Fluorinated ZIFs under Continuous Operational Conditions

Subsequently, several H_2_O intrusion/extrusion cycles were performed to evaluate the stability of the two fluorinated ZIFs under continuous operational conditions, up to 5 cycles for 24 h. In Figure 4C, intrusion volume (directly related with pore volume and, therefore, with integrity of the porous nature of the materials) is plotted versus cycles. A stable *V*_int_ value during successive cycles is observed for the two samples evaluated, in agreement with the high stability of standard ZIF-8. The study of the stability of the two fluorinated ZIFs was completed by means of PXRD by comparing the patterns before and after use. As shown in Figure 4D, the patterns of the two SOD-like fluorinated ZIFs are indistinguishable from those after cycling. This evidence confirms the structural stability of both fluorinated ZIFs under hydrostatic pressure after five intrusion/extrusion cycles. In contrast, the PXRD patterns of the pristine ZIF-8, both the commercial ZIF-8 and the as-prepared ZIF (Figure S11), revealed the appearance of some additional peaks (at around 11 and 19° in 2θ) after the water intrusion/extrusion cycles, which may be associated with degradation products, evidencing stability issues of nonfunctionalized ZIF-8 materials (even as-prepared ZIF) under the operational conditions. Note also that the contribution from an amorphous phase at around 11.5° was slightly larger in the case of commercial ZIF-8 compared to the as-prepared ZIF, most likely due to the presence of defects in the pristine commercial ZIF-8.

## 4 Conclusions

Overall, our results demonstrate that the fluorination of ZIFs can be a promising approach to finely modulate the mechanical energy behavior of ZIFs in HLSs in one direction or another (toward molecular-springs or shock-absorbers) by an appropriate and rationally designed functionalization of the MOF structure.

Moreover, we demonstrate that fluorination improved the chemical stability of the ZIF-structure not only in water and phosphate aqueous solutions but also under hydrostatic pressure when subjected to several water intrusion/extrusion cycles. It is expected that such an improvement has a positive effect on the stability of ZIFs under intrusion/extrusion cycling at higher temperatures, which currently is a limiting factor. It is also expected that fluorination affects the dynamic performance of mechanical energy dissipators under highly dynamic conditions. Both of these aspects will be addressed in upcoming works.

## Supporting Information Available

Additional experimental details and supporting data of the different evaluated ZIFs, e.g., NMR, refined XRD patterns, CIF files, adsorption isotherms, TEM, intrusion/extrusion data, etc. (PDF)
Crystallographic data for ZIF0 (CIF)
Crystallographic data for FZIF10 (CIF)
Crystallographic data for CF3ZIF10 (CIF)

## Supplementary Material

am4c09969_si_001.pdf

am4c09969_si_002.cif

am4c09969_si_003.cif

am4c09969_si_004.cif
