# Supplementary material for: Fluorinated Nanosized Zeolitic-Imidazolate Frameworks as Potential Devices for Mechanical Energy Storage
Source: ACS Appl Mater Interfaces. 2024 Aug 23;16(35):46374–83. doi: 10.1021/acsami.4c09969 (PMC11378149; doi:10.1021/acsami.4c09969)
Supplement: Supplementary file 1 — am4c09969_si_001.pdf [file am4c09969_si_001.pdf]

SUPPORTING INFORMATION

Fluorinated Nanosized Zeolitic-Imidazolate Frameworks as Potential Devices for Mechanical Energy Storage

Eder Amayuelas,[1] Judit Farrando-Perez,[2] Alexander Missyul,[3] Yaroslav Grosu,[1,4] Joaquin Silvestre-Albero[2,], Carolina Carrillo-Carrión[5,]**

[1] Centre for Cooperative Research on Alternative Energies (CIC energiGUNE), Basque Research and Technology Alliance (BRTA), 01510 Vitoria-Gazteiz, Spain.

[2] Laboratorio de Materiales Avanzados, Departamento de Química Inorgánica-Instituto Universitario de Materiales, Universidad de Alicante, 03690 San Vicente del Raspeig, Spain. E-mail: joaquin.silvestre@ua.es

[3] CELLS – ALBA Synchrotron, Cerdanyola del Vallés, Barcelona, Spain.

[4] Institute of Chemistry, University of Silesia, 40-006 Katowice, Poland.

[5] Institute for Chemical Research (IIQ), CSIC-University of Seville, 41092 Sevilla, Spain. E-mail: carolina.carrillo@csic.es

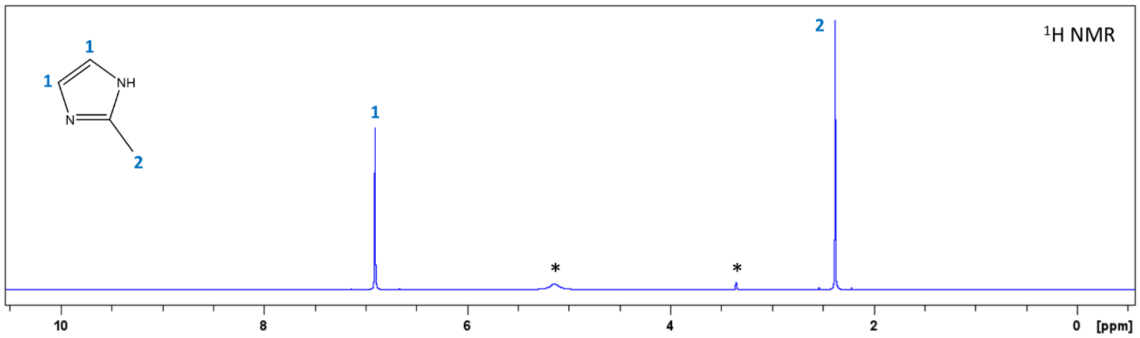

Figure S1. ^{1}H NMR spectrum recorded at 400 MHz in MeOD of the 2-methylimidazole (MeImz). Peaks indicated with * come from the solvents.

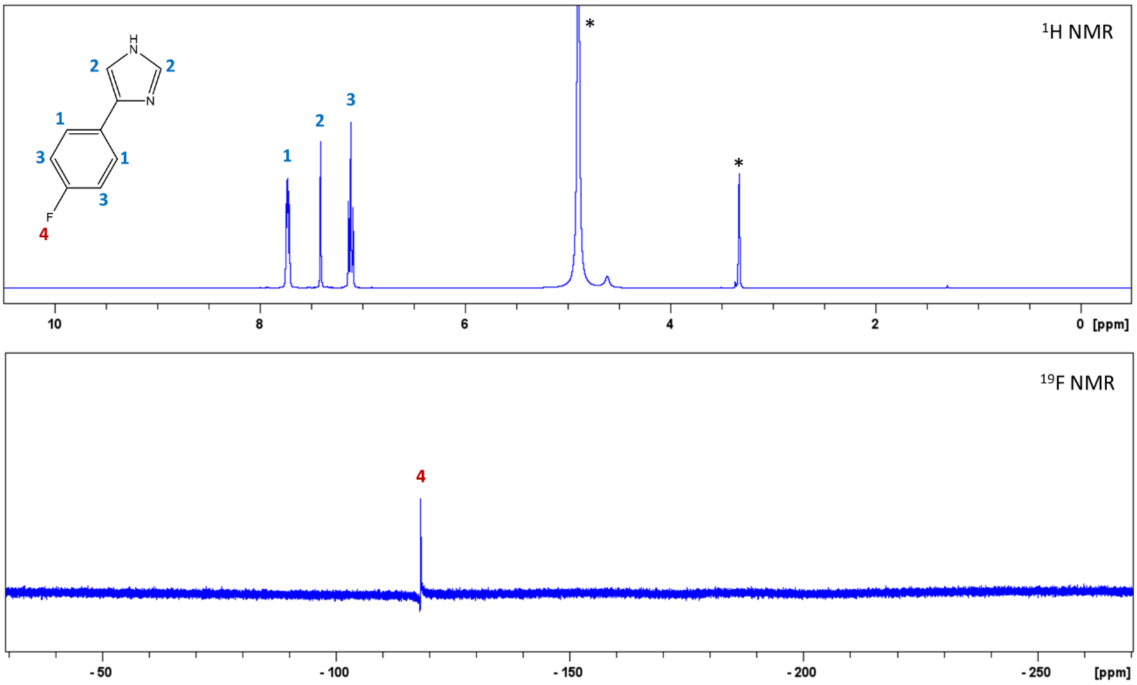

Figure S2. ^{1}H NMR and ^{19}F NMR spectra recorded at 400 MHz in MeOD of the 4-(4-fluorophenyl)-1H-imidazole (FImz). Peaks indicated with * come from the solvents.

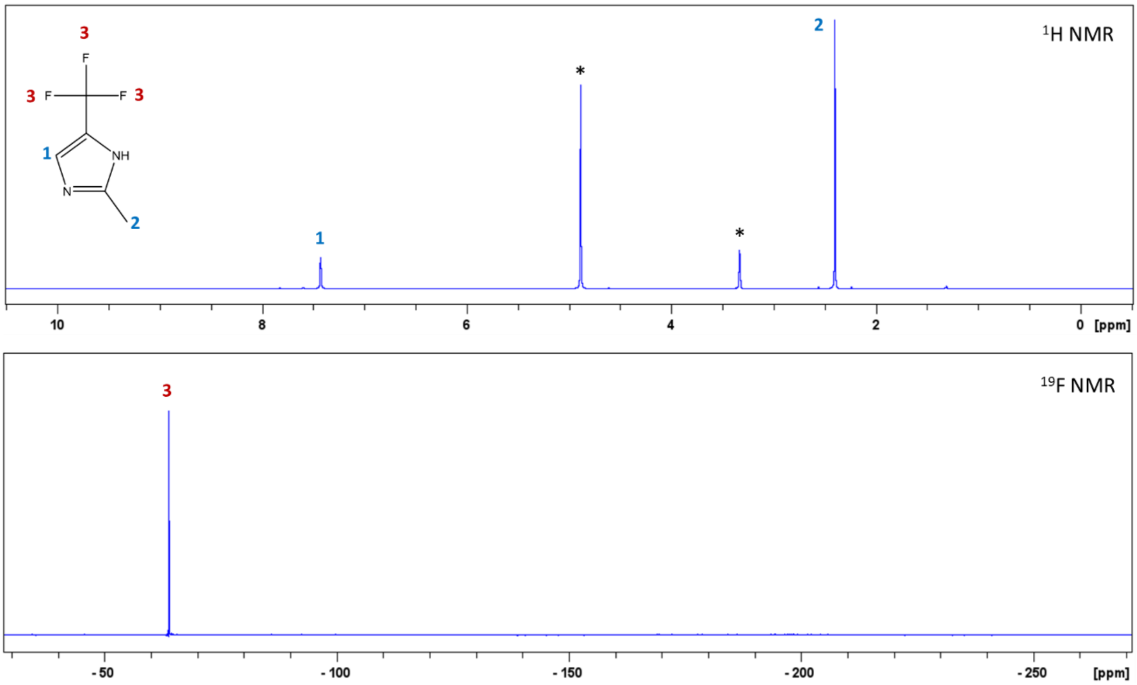

Figure S3. ^{1}H NMR and ^{19}F NMR spectra recorded at 400 MHz in MeOD of the 2-methyl-5-(trifluoromethyl)-1H-imidazole (CF3Imz).

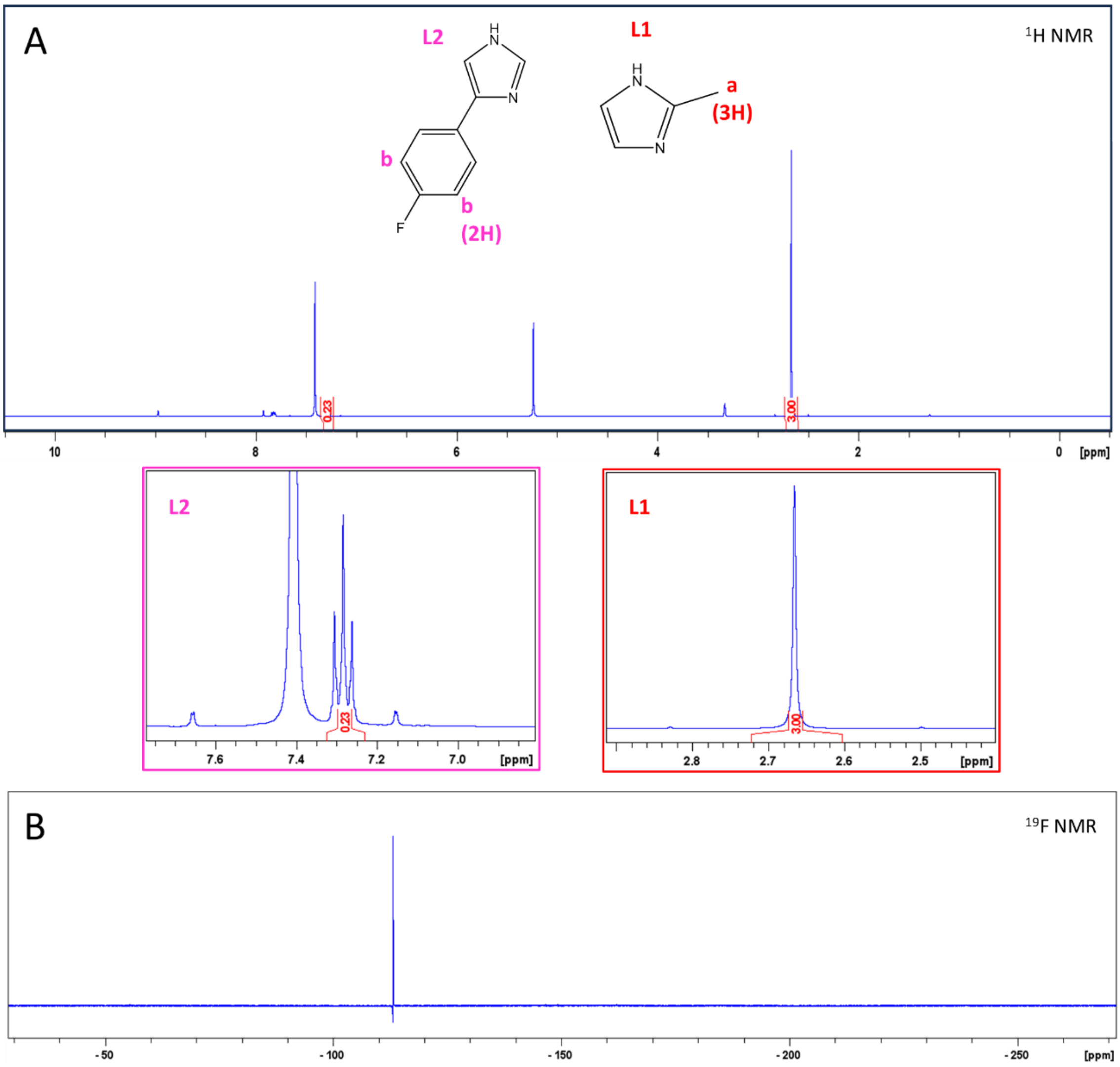

Figure S4. (A) ^{1}H NMR spectrum (400 MHz) of the acid digested FZIF10 sample (prepared by adding a 10% of the linker L2 in the precursors solution). Experimental ratio L1: L2 = 9.0: 1.03 (theoretical ratio = 9.0: 1.0). (B) ^{19}F NMR spectrum (400 MHz) of the acid digested FZIF10 sample.

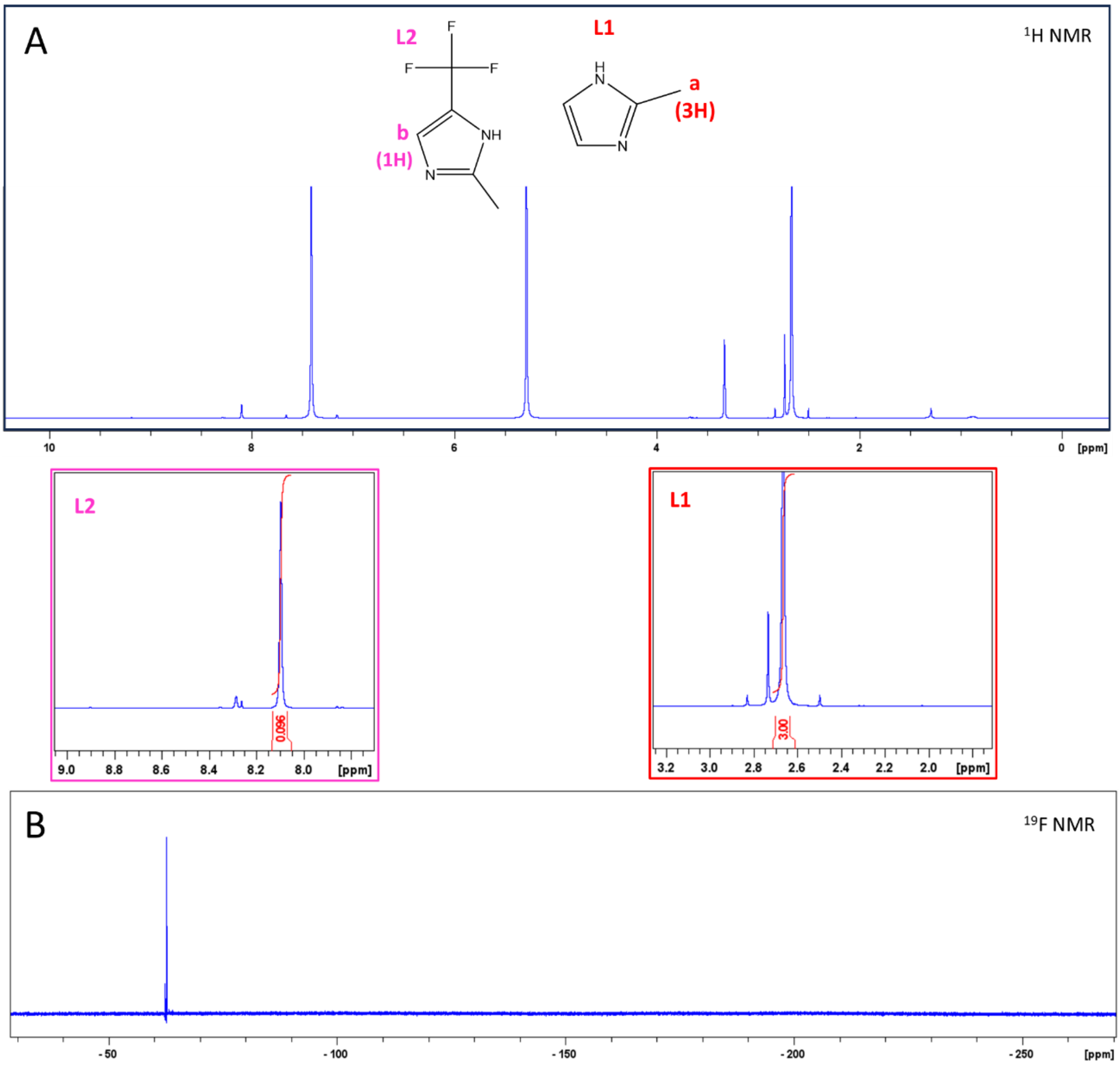

Figure S5. (A) ^{1}H NMR spectrum (400 MHz) of the acid digested CF3ZIF10 sample (prepared by adding a 10% of the linker L2 in the precursors solution). Experimental ratio L1: L2 = 9.0: 0.96 (theoretical ratio = 9.0: 1.0). (B) ^{19}F NMR spectrum (400 MHz) of the acid digested CF3ZIF10 sample.

Table S1. Size of dried particles expressed in diameter d (mean value ± SD; particles idealized as spherical) as determined by SEM images, and hydrodynamic diameters d_h (mean value ± SD) as derived from DLS size distributions of the particles freshly dispersed in either methanol or MilliQ water. Polydispersity index (PdI) is also given.

		MeOH		H_2O	
**Sample**	**d (nm ± SD)[a]**	**d_h (nm ± SD)[b]**	**PdI**	**d_h (nm ± SD)[b]**	**PdI**
ZIF	89 ± 10	111.2 ± 2.2	0.143	108.2 ± 3.5	0.185
FZIF10	217 ± 23	233.4 ± 4.6	0.203	252.3 ± 5.1	0.215
CF3ZIF10	96 ± 8	114.7 ± 2.1	0.121	132.0 ± 3.6	0.136

(a) SEM images analysis (N=100); (b) DLS size distributions by number.

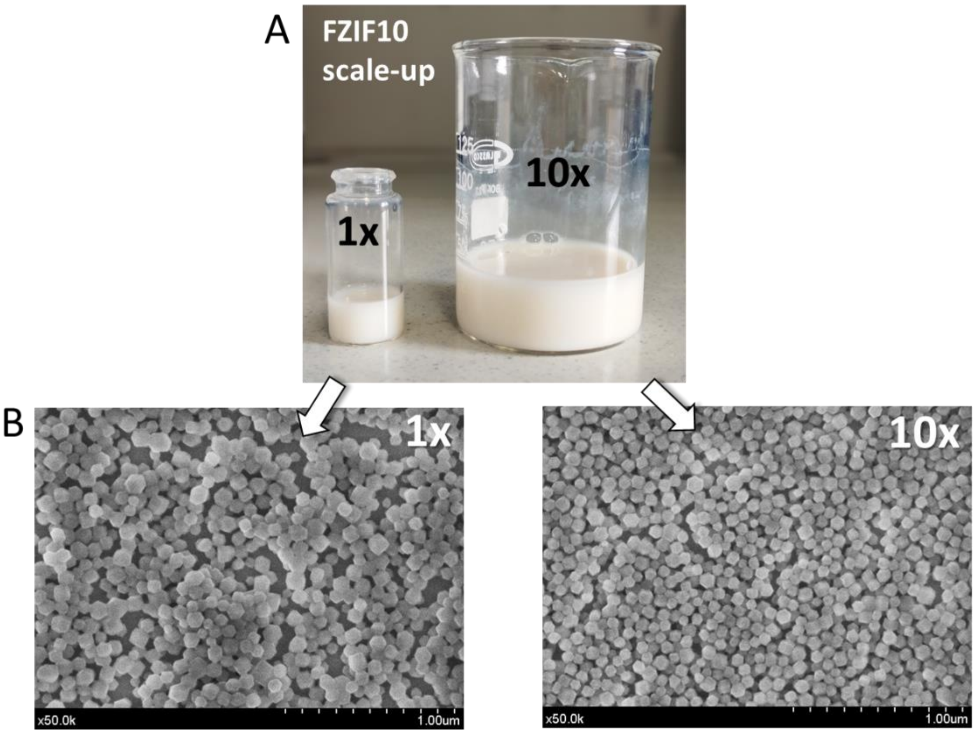

Figure S6. Scaling-up the FZIF10 synthesis. (A) Photographs of the FZIF10 particles prepared in small-scale (1x) that yielded 12 mg of particles (88% yield), and large-scale (10x) that yielded a total of 119 mg of material (86.4% yield). (B) SEM images of the particles obtained under small- and large-scale.

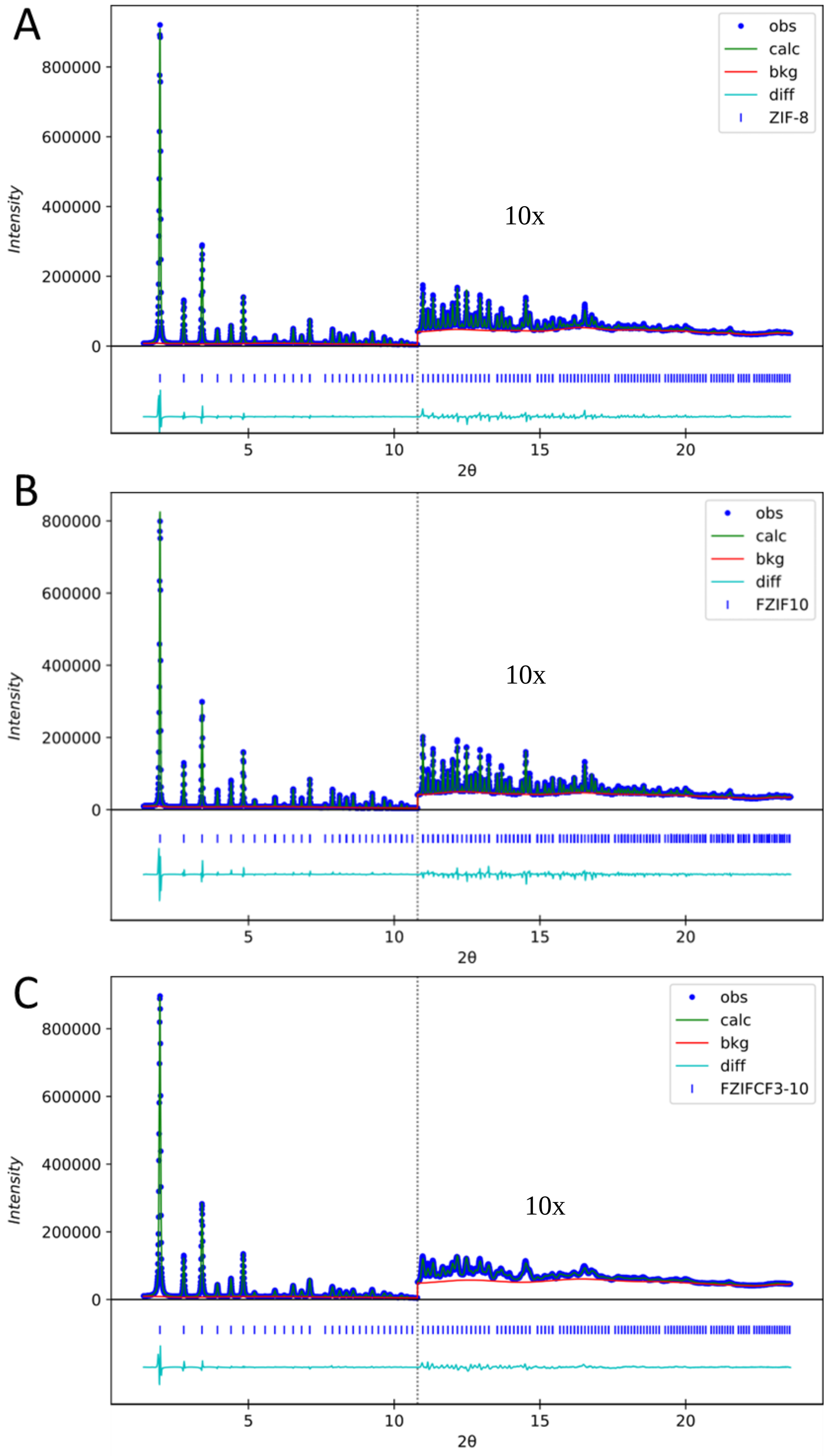

Figure S7. Rietveld refinement of the as-synthetised samples: (A) ZIF, (B) FZIF10 and (C) CF3ZIF10 particles. Right hand side of the SXRPD patterns were 10x amplified.

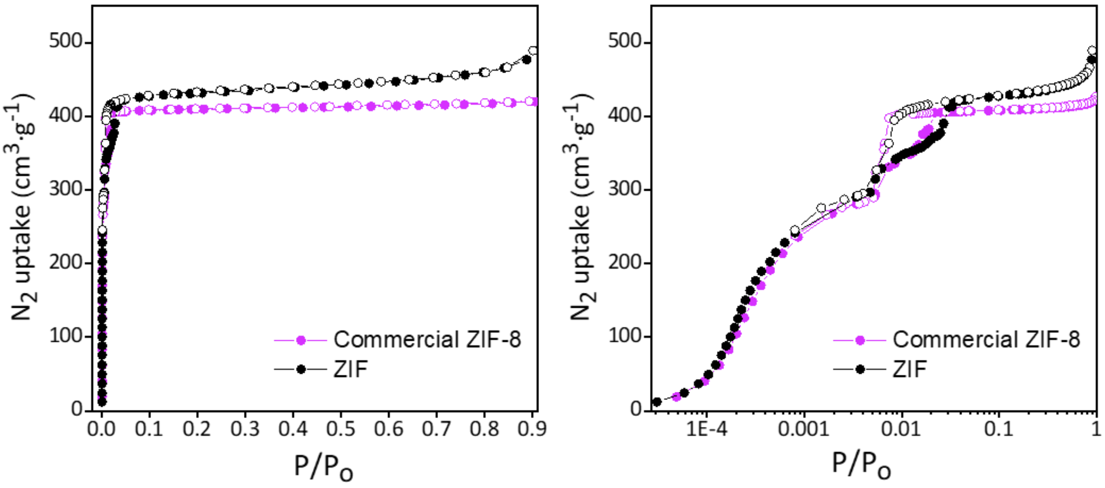

Figure S8. N_2 adsorption/desorption isotherms at -195ºC for the pristine ZIF and the commercial ZIF-8 (Basolite® Z1200) sample in linear (right) and logarithmic (left) scale.

Table S2. Textural properties of the different particles as determined from N_2 isotherms.

Sample	S_{BET} ($m^2\ g^{-1}$)	V_{total} ($cm^3\ g^{-1}$)	V_{micro} ($cm^3\ g^{-1}$)	$V_{total-micro}$ ($cm^3\ g^{-1}$)
Commercial ZIF-8	1570	0.66	0.63	0.03
ZIF	1678	0.76	0.67	0.09
FZIF10	1327	0.58	0.50	0.08
CF3ZIF10	1545	0.71	0.58	0.13

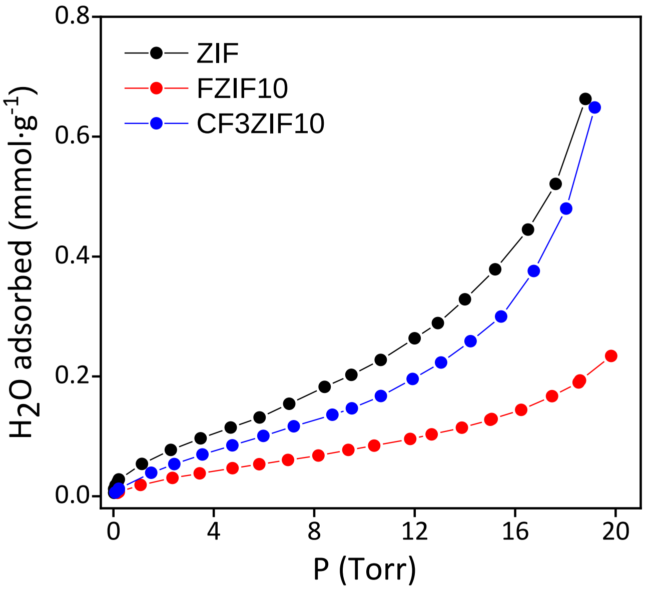

Figure S9. H_2O adsorption isotherms at 25ºC for the as-prepared ZIFs.

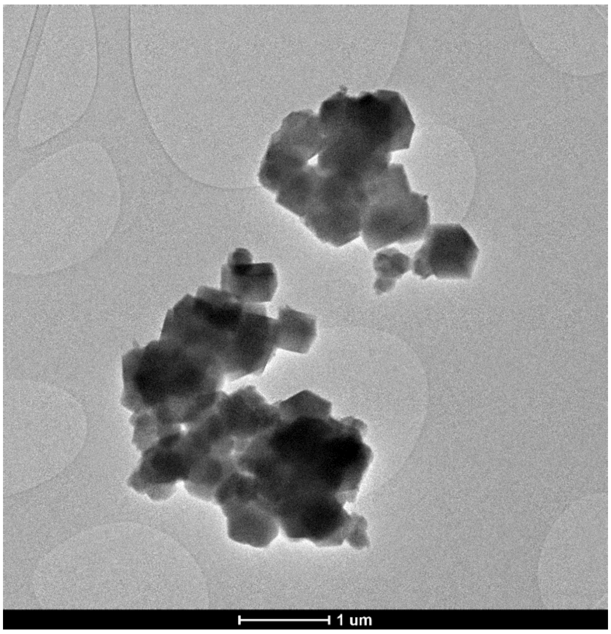

Figure S10. TEM image of commercial ZIF-8 (Basolite® Z1200) for comparison of the size and quality of the particles with respect to the as-prepared control ZIF particles.

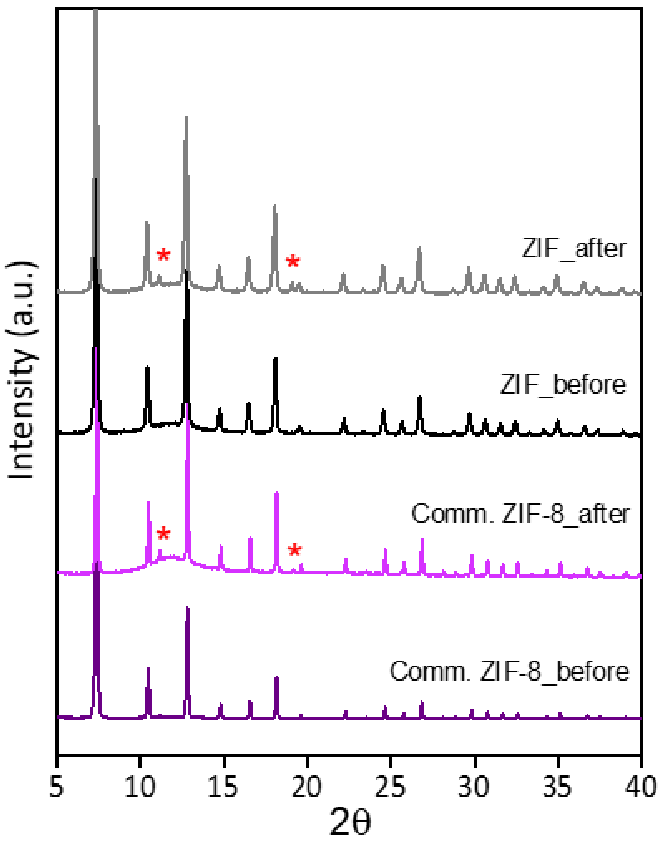

Figure S11. PXRD patterns of the commercial ZIF-8 and the as-prepared ZIF before and after the five H_2O intrusion-extrusion cycles. Asterisks highlight the appearance of degradation products.

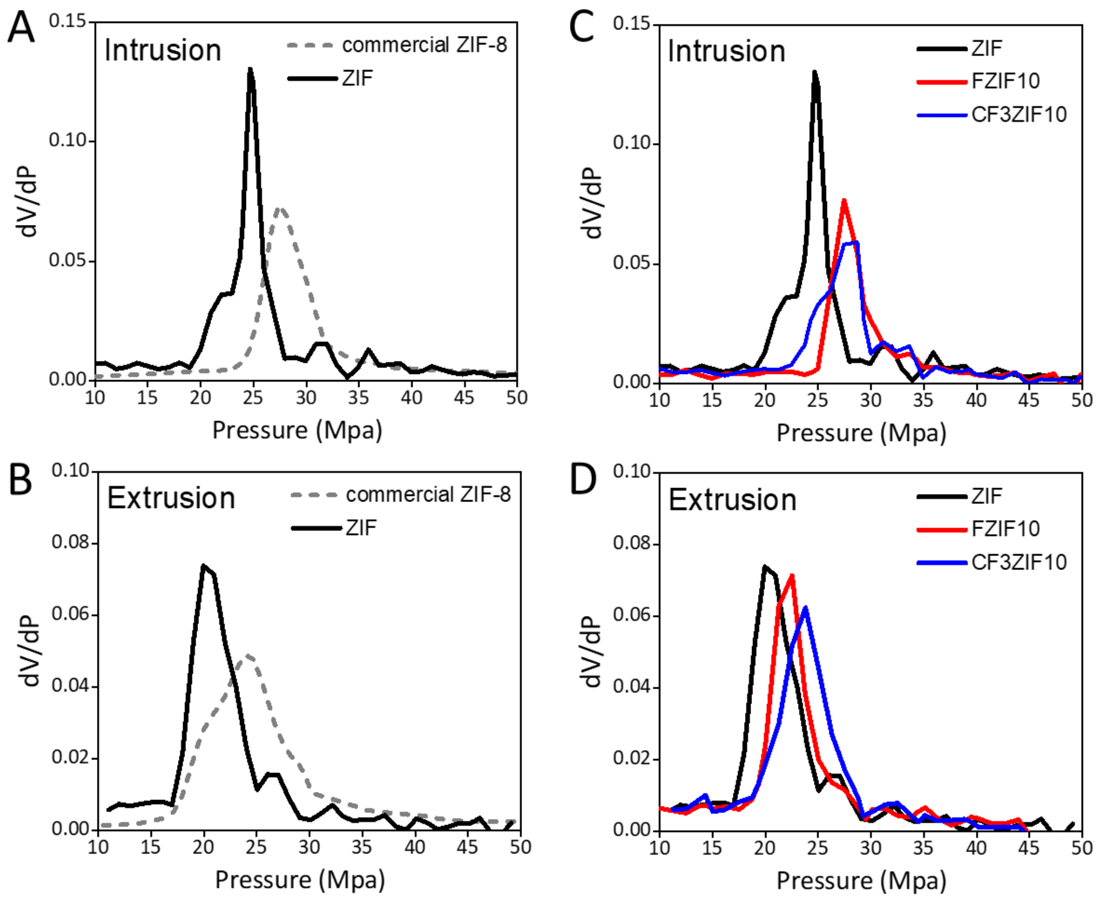

Figure S12. (A-B) Derivatives of the intrusion and extrusion branches of *PV* isotherms for the as-prepared ZIF (black solid lines) and the commercial ZIF-8 sample (grey dotted lines). (C-D) Derivatives of the intrusion and extrusion branches of *PV* isotherms for the as-prepared ZIF (black lines), FZIF10 (red lines) and CF3ZIF10 (blue lines).
